# Electrochemical removal of pyrite scale using green formulations

**DOI:** 10.1038/s41598-021-84195-9

**Published:** 2021-02-26

**Authors:** Musa Ahmed, Ibnelwaleed A. Hussein, Abdulmujeeb T. Onawole, Mohammed A. Saad, Mazen Khaled

**Affiliations:** 1 Gas Processing Center, College of Engineering, Qatar University, P.O. Box 2713, Doha, Qatar; 2 Chemical Engineering Department, College of Engineering, Qatar University, P.O. Box 2713, Doha, Qatar; 3 Department of Chemistry and Earth Sciences, Qatar University, P.O. Box 2713, Doha, Qatar

**Keywords:** Electrochemistry, Materials chemistry

## Abstract

Pyrite scale formation is a critical problem in the hydrocarbon production industry; it affects the flow of hydrocarbon within the reservoir and the surface facilities. Treatments with inorganic acids, such as HCl, results in generation toxic hydrogen sulfide, high corrosion rates, and low dissolving power. In this work, the dissolution of pyrite scale is enhanced by the introduction of electrical current to aid the chemical dissolution. The electrolytes used in this study are chemical formulations mainly composed of diethylenetriamine-pentaacetic acid–potassium (DTPAK_5_) with potassium carbonate; diethylenetriamine pentaacetic acid sodium-based (DTPANa_5_), and l-glutamic acid-*N*, *N*-diacetic acid (GLDA). DTPA and GLDA have shown some ability to dissolve iron sulfide without generating hydrogen sulfide. The effect of these chemical formulations, disc rotational rate and current density on the electro-assisted dissolution of pyrite are investigated using Galvanostatic experiments at room temperature. The total iron dissolved of pyrite using the electrochemical process is more than 400 times higher than the chemical dissolution using the same chelating agent-based formulation and under the same conditions. The dissolution rate increased by 12-folds with the increase of current density from 5 to 50 mA/cm^2^. Acid and neutral formulations had better dissolution capacities than basic ones. In addition, doubling the rotational rate did not yield a significant increase in electro-assisted pyrite scale dissolution. XPS analysis confirmed the electrochemical dissolution is mainly due to oxidation of Fe^2+^ on pyrite surface lattice to Fe^3+^. The results obtained in this study suggest that electro-assisted dissolution is a promising technique for scale removal.

## Introduction

Pyrite (FeS_2_) is also known as the fool’s gold due to its similarity with gold in appearance, which earned its gangue nature^1,2^, is presumed to be the most common sulfide mineral. Hence, it is of little surprise that it is often found when mining for other valuable minerals, such as gold^3^. This gangue nature of pyrite has led to the development of several processes in separating pyrite from other minerals. Oxidative leaching and anodic dissolution are examples of hydrometallurgical methods used in destroying pyrite structure and uncovering gold particles^4,5^. These applications are applied in metal extraction and acid mine drainage^6^. Pyrite is also removed via oxidative dissolution in coal-powered stations to reduce the level of sulfur dioxide emission. However, besides the mining industry, pyrite causes another problem in the oil and gas industry as it forms scales in reservoirs. These scale deposits hinder the flow assurance near-wellbore area of the reservoir, which consequently leads to blockage of the downhole tubular, formation damage, and complete shutdown of production and operational processes.

Both mechanical and chemical methods were applied in resolving the problems created by pyrite scales. However, the latter is more popular than the former since mechanical methods involve physical removal of the scale, which often aggravates the situation by enhancing corrosion. Whereas, chemical methods, which include the use of chelating agents, have proved useful in removing iron sulfide scales which have about equal ratio of iron to sulfur atoms such as pyrrhotite (Fe_7_S_8_) and troilite (FeS)^7–9^. Nevertheless, pyrite scale is quite stubborn even for chelating agents and this is due to the high ratio of sulfur to iron (2:1), which enhances its stability. Hence, the difficulty in removing pyrite scale among other iron sulfide scales.

In our previous works^10,11^, we have applied both experimental and computational methods in studying the chemical removal of iron sulfides using green materials including different chelating agents . Unlike other chemical methods that use corrosion inhibitors for removing scales, no toxic gases such as H_2_S are generated^7,12^ as shown in Eq. (1). There is a need for better processes such as electrochemical methods, which would offer a greener and effective solution for pyrite scale removal in oil and gas wells.

1 $${\text{FeS }} + {\text{ 2HCl}} \to {\text{ H2S }} \uparrow + {\text{FeCl}}_{{2}}$$

Electrochemical methods including anodic dissolution have many applications in the mining industry as well as the oil and gas industry. The use of redox potential has been shown to aid pyrite oxidation which has its application in acid mine drainage^13^. A recent study reported that the oxidation of pyrite is important in black shale oxidation process which is also relevant to the oil and gas industry^14^. Pyrite oxidation is the initial step in the dissolution of pyrite structure. Previous studies have shown that Fe^3+^ and dissolved oxygen are the essential oxidants involved in pyrite dissolution process^15–17^; Li et al.^15,18^ proposed the mechanism shown in Eqs. (2)–(4). Efforts have been made to determine the ratio of ferric to ferrous, which is often done by measuring the redox potential and also the regeneration of ferric ions from ferrous ions as shown in Eq. (2). Hence, redox reaction plays a predominant role in the oxidative pyrite dissolution^15^. Iron ions redox reactions have been studied recently by Li et al.^4,15^.

2 $$2{FeS}_{2}+2{H}_{2}O+7{O}_{2} {\to 2Fe}^{2+}+{4SO}_{4}^{2-}+4{H}^{+}$$

3 $$4{Fe}^{2+}+{O}_{2}+4{H}^{+} \to {4Fe}^{3+}+2{H}_{2}O$$

4 $${FeS}_{2}+8{H}_{2}O+{14Fe}^{3+} \to 15{Fe}^{2+}+{2SO}_{4}^{2-}+16{H}^{+}$$

In some reactions, the rate-determining step in the propagation stage of pyrite oxidation is reported to be the oxidation of Fe^2+^ to Fe^3+^^19^. Under electrochemical dissolution, numerous investigations found that electrochemical processes control the rate of pyrite oxidation and dissolution^20–23^. In addition Garrels and Thompson^24^ also found that pH does not affect significantly the pyrite oxidation rate in the range of pH 0–2, which maybe significant at higher pH where Fe(OH)_3_ might form as an additional oxidation product. For the oxidation reaction:

5 $${\text{FeS}}_{{2}} \to {\text{Fe}}^{{2+}} + {\text{2S}}^\circ + {\text{2e}}$$

A full electrochemical mechanism is presented in^25^ while a recent study^26^ showed that at low pH there are two different pathways for the pyrite oxidation. The low voltage region, around 0.5 vs Ag/AgCl, and characterized by the formation of a surface layer of Fe(OH)_3_ and S_0_ which covers the surface and resulted in passivation. At higher voltages Fe_2_O_3_, FeO and S_8_ would form and as it accumulated on the pyrite surface, electrochemical oxidation became difficult even at higher potentials.

In this paper, in order to investigate new green formulations to prevent the formation of H_2_S usually associated with the anodic oxidation of pyrite at low pH, electrochemical dissolution (oxidation) of pyrite is studied by carrying out Galvanostatic experiments (application of a constant current density by a potentiostat), using a rotating disk electrode (RDE). The effect of chemical formulations, disc rotational rate and current density on pyrite dissolution are investigated. Unlike other chemical methods of scale removal, the proposed method is hydrogen sulfide free as no rotten egg smell confirming its presence was observed. Besides, this study emphasizes the role of electrochemical techniques in providing a major enhancement in the dissolution of pyrite scale using green formulations.

## Experimental

### Materials and solutions

**Figure 1:**
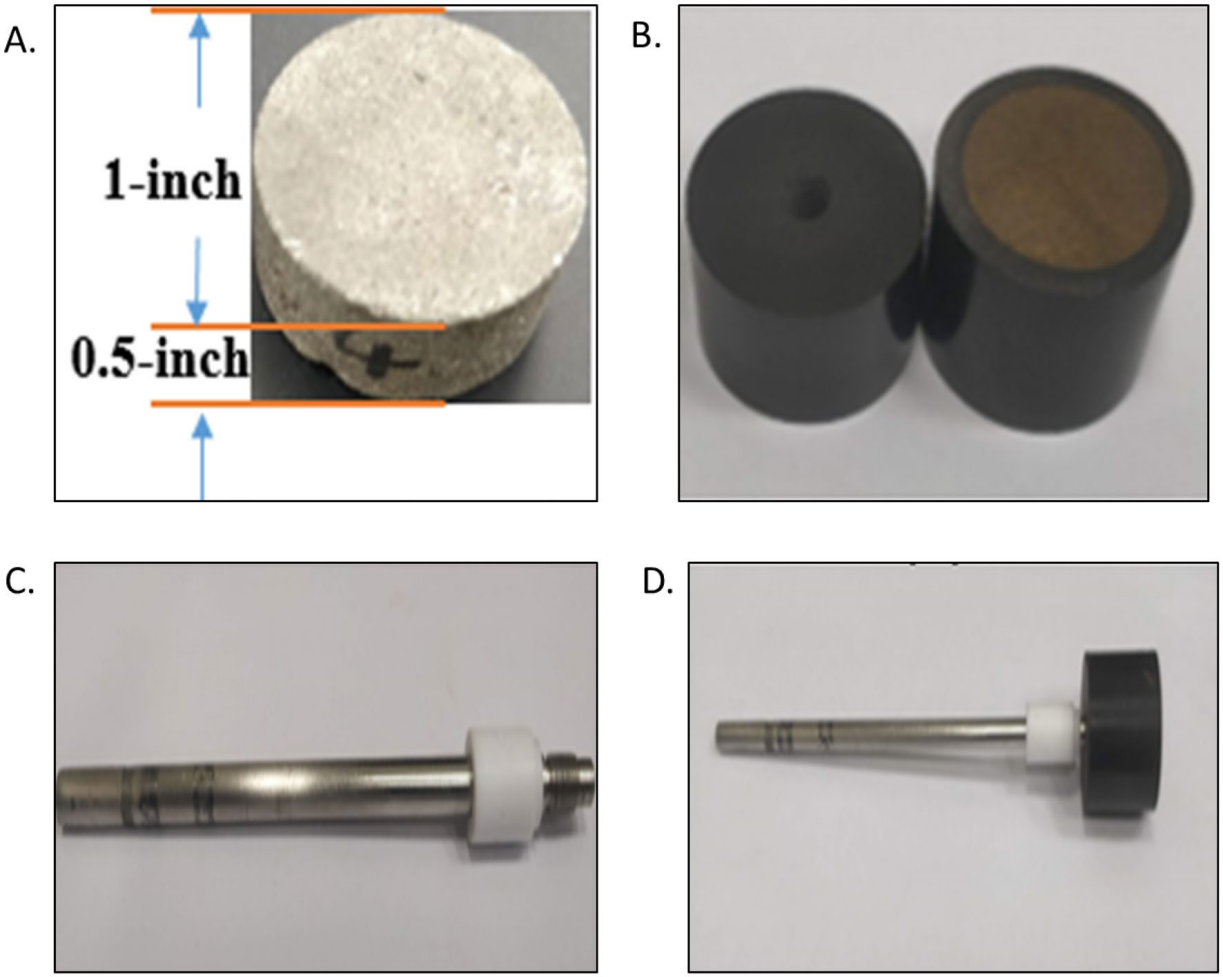
The **(a)** pyrite disk electrode with its dimension, **(b)** the insulator with the casted pyrite electrode, **(c)** rotating disk electrode (RDE) shaft, **(d)** coupled RDE shaft with pyrite electrode.

Pyrite with high purity was used in this study. Geological Store Company, England supplied the samples in the form of large crystal rocks. The pyrite rock has measured porosity of 0.05%. Chemical analysis for these samples, published elsewhere^27^, shows that it contains more than 99% pyrite with some impurities of Si, Na, Ca, Mg and Al. The working electrode of pyrite was prepared by drilling core samples of 1-in diameter and 0.5-inch thickness disks as shown in Fig. 1A. After that, one of the two circular surfaces was highly polished using sandpapers with different grit sizes. Then, all the disk surfaces were casted and covered using phenolic resin except the polished surface as in Fig. 1B. A small thread hole was drilled through the back of the electrode holder, penetrating some mm inside the pyrite disk (Fig. 1B). The diameter of those threads was machined to fit the RDE shaft thread. Electrical contact was established by tightening the RDE shaft (Fig. 1C; male part) to the threaded pyrite electrode (Female part; Fig. 1D). A renewed surface was created on the electrode preceding to each experiment through hand polishing consecutively using papers of silicon carbide with different roughness. After washing thoroughly with deionized water, the electrode was directly submerged in the electrolyte.

### Chemical solutions

The green formulations that are recently reported as an effective dissolver for pyrite were used as the electrolyte in this study^8,27^. The chemical dissolution results obtained from our previous work was compared with the electrochemical dissolution data obtained here. These formulations are green in the sense that they dissolve the pyrite scale without generating H_2_S as no rotten egg smell was perceived during the whole experiment. Different concentrations of chelating agents and salts were used. The green formulations include borax, diethylenetriamine-pentaacetic acid–potassium (DTPAK_5_) with potassium carbonate, diethylenetriamine pentaacetic acid sodium-based (DTPANa_5_), and L-glutamic acid-N, N-diacetic acid (GLDA). AkzoNobel Company, Netherlands, provided the chelating agents used in this study. All solutions were prepared with ACS reagent grade chemicals and deionized water. The chemical formulations used and the initial pH of the solutions are shown in Table 1.

**Table 1:** Chemical formulations used in the anodic dissolution experiment.

#	Chemical formulation	Initial pH
1	DTPAK_5_ 10 wt.%	13.23
2	DTPAK_5_ 10 wt.% + K_2_CO_3_ 9 wt.%	13.49
3	DTPAK_5_ 10 wt.% + K_2_B_4_O_7_ 6 wt.%	6.77
4	DTPAK_5_ 10 wt.% + K_2_B_4_O_7_ 9 wt.%	10.5
5	DTPANa_5_ 10 wt.%	12.74
6	GLDA 10 wt.%	3.6
7	GLDA 5 wt.% + DTPAK_5_ 5 wt.%	7.64

### Experimental set up

**Figure 2:**
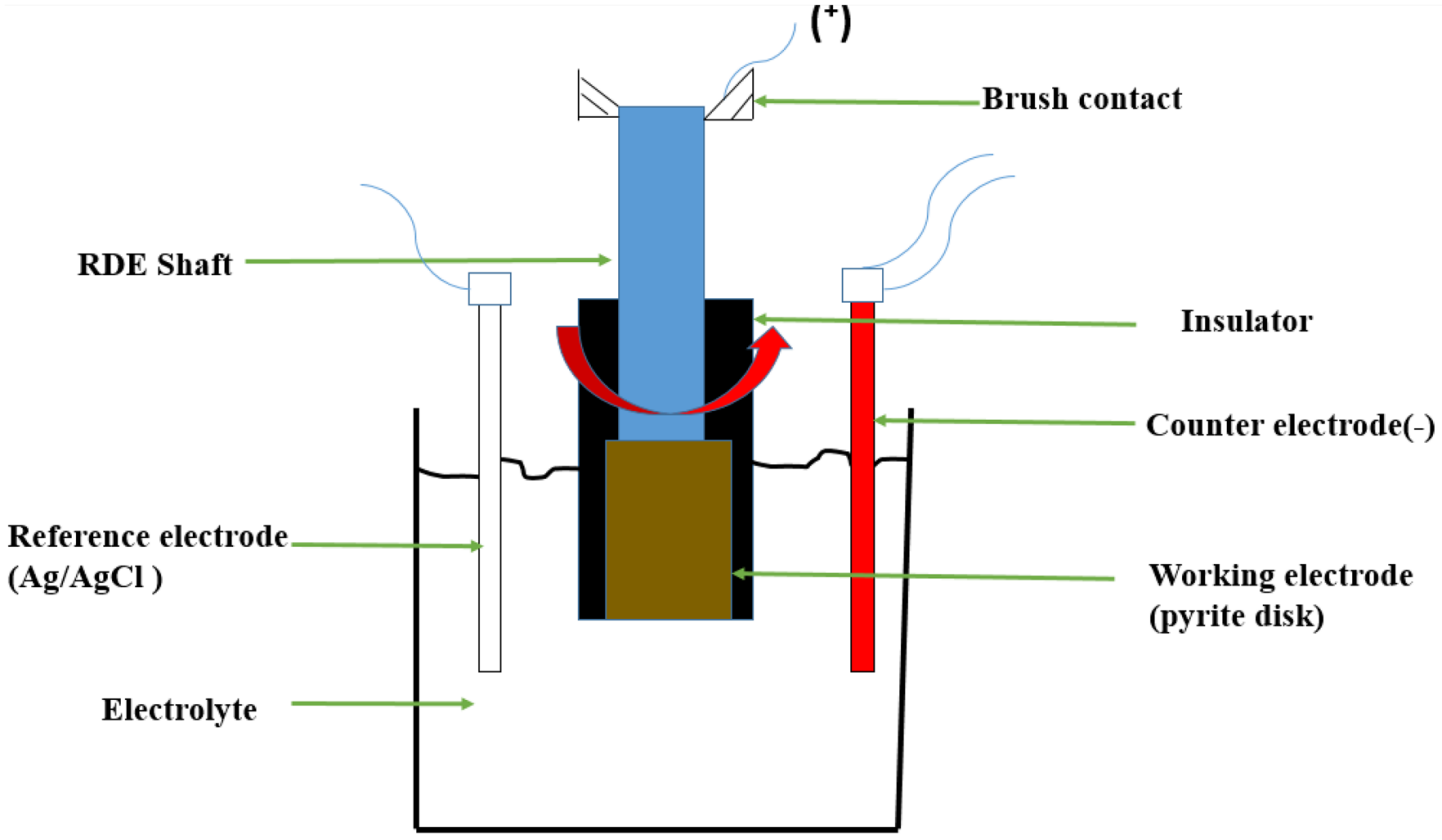
Schematic of the 3-electrode system used in this study.

For the electrochemical measurements, a 3-electrode cell was used here as it provides the material's electrochemical signature (pyrite only here). A typical 3-electrode electrochemical cell (Fig. 2) with a 400 ml beaker is used for Galvanostatic tests. The pyrite sample, illustrated in Fig. 1, was used as a working electrode with a surface area of 5.07 cm^2^. In addition, a reference electrode made of silver/silver chloride (Ag/AgCl) filled with 3 M KCl solution was used and placed inside a Luggin capillary filled with the test solution to minimize Ohmic drop. The third electrode was the counter electrode, which is made from 6 mm diameter graphite. All electrochemical experiments were performed on Gamry Interface 1010E Potentiostat (± 0.3% accuracy on potential and current readings) using Gamry framework data acquisition software (version 7.06) to control the instrument and record potential changes while applying constant current density. Echem Analysts Software was used to analyze the data. Iron concentration in the solution was determined through ICP-OES measurements.

### Experimental methodology

**Figure 3:**
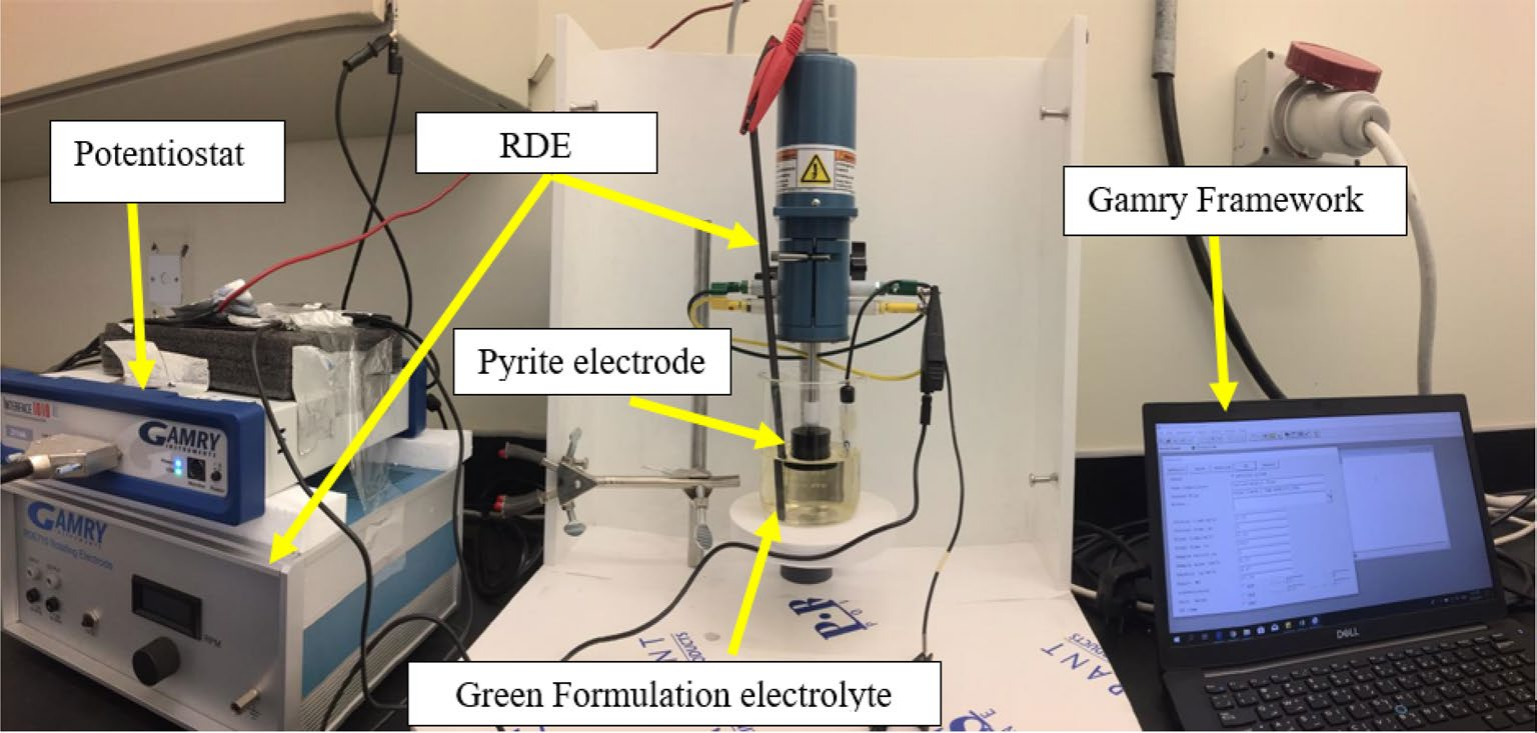
The Potentiostat and the rotating disc electrode (RDE).

The rotating disk electrode (RDE) was scuffed with a sequence of sandpapers with different roughness, followed by cleaning in distilled water before drying. Then, the RDE is immersed in a 200 ml solution of the green formulation, which was prepared with the desired concentration for each test. Constant current density tests (Fig. 3) were applied to examine the effect of the applied current on the dissolution rate of pyrite. All Galvanostatic tests were executed at two current densities 5 mA/cm^2^ and 50 mA/cm^2^. The current densities were selected after some random trial experiments with a wide range of current densities from 0.05 mA/cm^2^ up to 500 mA/cm^2^ were carried out. Current density higher than 50 mA/cm^2^ leads to overload, while at a current density below 5 mA/cm^2^ there are no significant changes in the dissolution of pyrite. The basis of the guided current experiments is that a redox reaction (electron transfer) must occur on the surface of the working electrode to sustain the applied current. All the electrochemical measurements were performed at room temperature and atmospheric pressure at different disk rotational rate (revolutions per minute, rpm) for 30 min under constant current density. After each experiment, the iron concentration in the solution was measured using ICP-OES. Before ICP measurement the aqueous samples were diluted 100 times to reduce the cation and hydrocarbon concentration to the acceptable limit for measurements. The total dissolved iron is divided by surface area and time to get the dissolution rate in ppm/cm^2^-s. Finally, X-ray photoelectron spectroscopy (XPS) and high-resolution images for the electrode surface were obtained to help with the overall understanding of the electrochemical reaction.

### Surface observation and composition analysis

The surface characteristic of the pyrite sample before and after the Galvanostatic tests were detected using XPS. Some samples that represent different pH solutions were selected. A high-resolution camera was also used for visual observation of the pyrite electrode specimens as well as for the identification of chemical changes that occurred during experiments.

## Results and discussion

### Comparison of chemical and electrochemical dissolution

**Figure 4:**
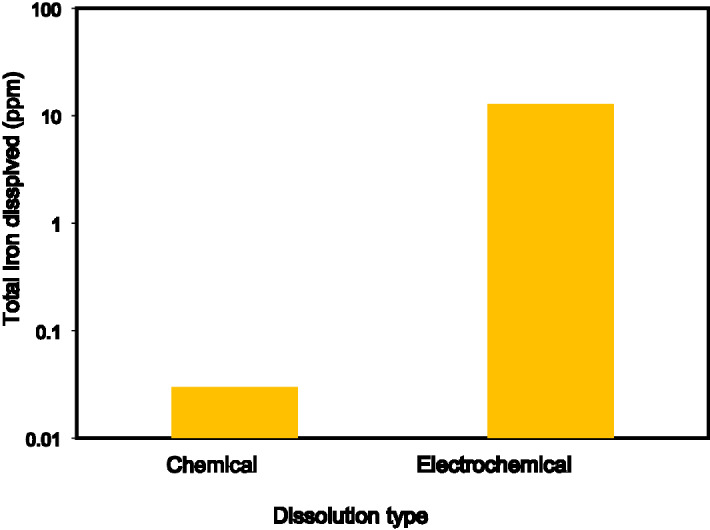
Comparison of total iron dissolved in chemical and electrochemical dissolution in DTPA/K_2_CO_3_ solution at room temperature.

**Figure 5:**
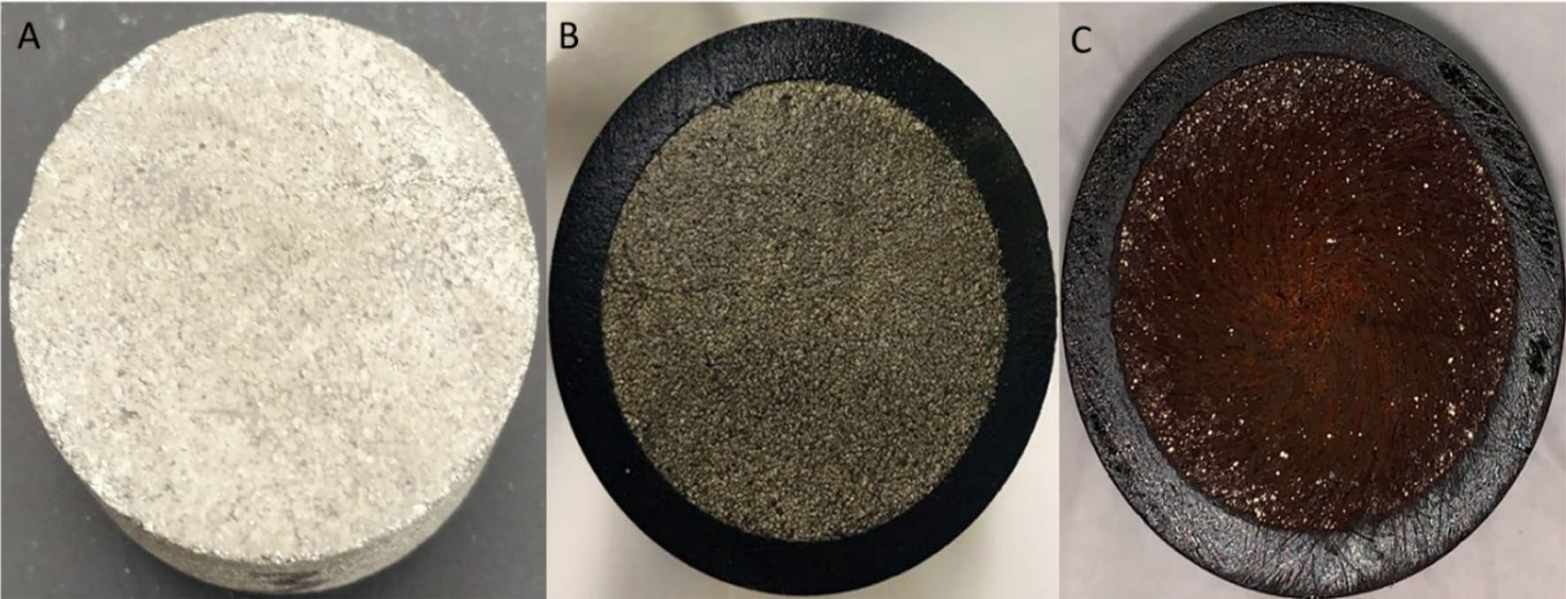
Pyrite samples: **(A)** before reaction **(B)** after chemical reaction with DTPA/K_2_CO_3_ solution **(C)** after electrochemical reaction in DTPA/K_2_CO_3_ solution.

The chemical and the electrochemical dissolution was performed in the system of RDE and Potentiostat. In the case of a chemical dissolution, the Potentiostat was switched off and no current was applied, whereas, in the electrochemical experiment a constant current density of 50 mA/cm^2^ was applied at the working electrode. Both experiments were carried out at room temperature, atmospheric pressure, and 1000 rpm for 30 min using a green formulation of DTPA/K_2_CO_3_. DTPA/K_2_CO_3_ is used because it serves as the reference point as it was the first green formulation developed in our lab^8^. The results of iron concentration from ICP measurement, under the same conditions, show that total iron dissolved from the electrochemical dissolution is about more than 400 times higher than that of the chemical dissolution at the same conditions, as shown in Fig. 4. This indicates that the electrochemical dissolution could make a significant boost to the technology of pyrite scale removal. In Fig. 5, a noteworthy change of the pyrite surface was observed and this is mainly due to pyrite oxidation in the presence of oxygen when the current is applied (Fig. 5C).

### Effect of different formulations on pyrite dissolution

Different green formulations were evaluated to study their potential use as an electrolyte for more efficient pyrite dissolution. These electrolytes were composed from the components of green formulations were used in our previous works for dissolving pyrite scale^8,27^. Chelating agents and/or salts were the main components of these formulations. The chelating agents that are commonly used for iron sulfide chemical removal were selected, namely, DTPANa_5_, DTPAK_5_, and GLDA. Potassium carbonate, K_2_CO_3_, and potassium tetra borate, K_2_B_4_O_7_-4H_2_O salts were added to the chelating agents looking for synergistic effects that maximize the pyrite dissolution as reported in one of our previous work^8^. Using both theoretical^11^ and experimental^10^ techniques, it was observed that addition of chemicals such as K_2_B_4_O_7_–4H_2_O (borax) are responsible for the dissolution of the pyrite scale and leading to available free Fe^2+^ ions which are then easily captured by the chelating agents. These chemicals break the pyrite scale by using the potassium ions present in them to bind to the sulfur atoms in pyrite there to form K-S bonds. The experiments in this section were carried out at atmospheric pressure, room temperature, a current density of 5 mA/cm^2^, for 30 min.

**Figure 6:**
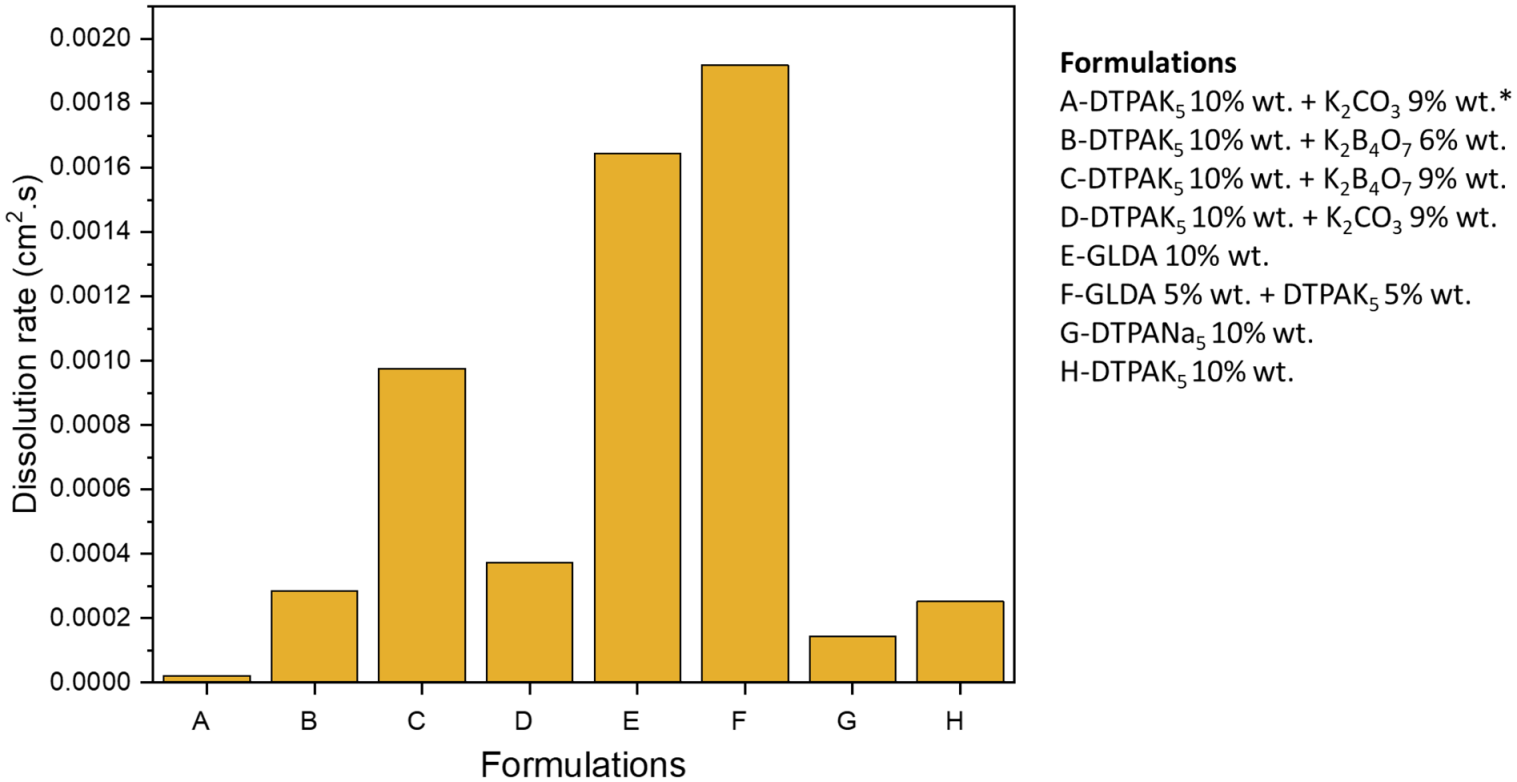
Effect of chemical formulations on the dissolution rate of pyrite. * indicates no current density.

**Figure 7:**
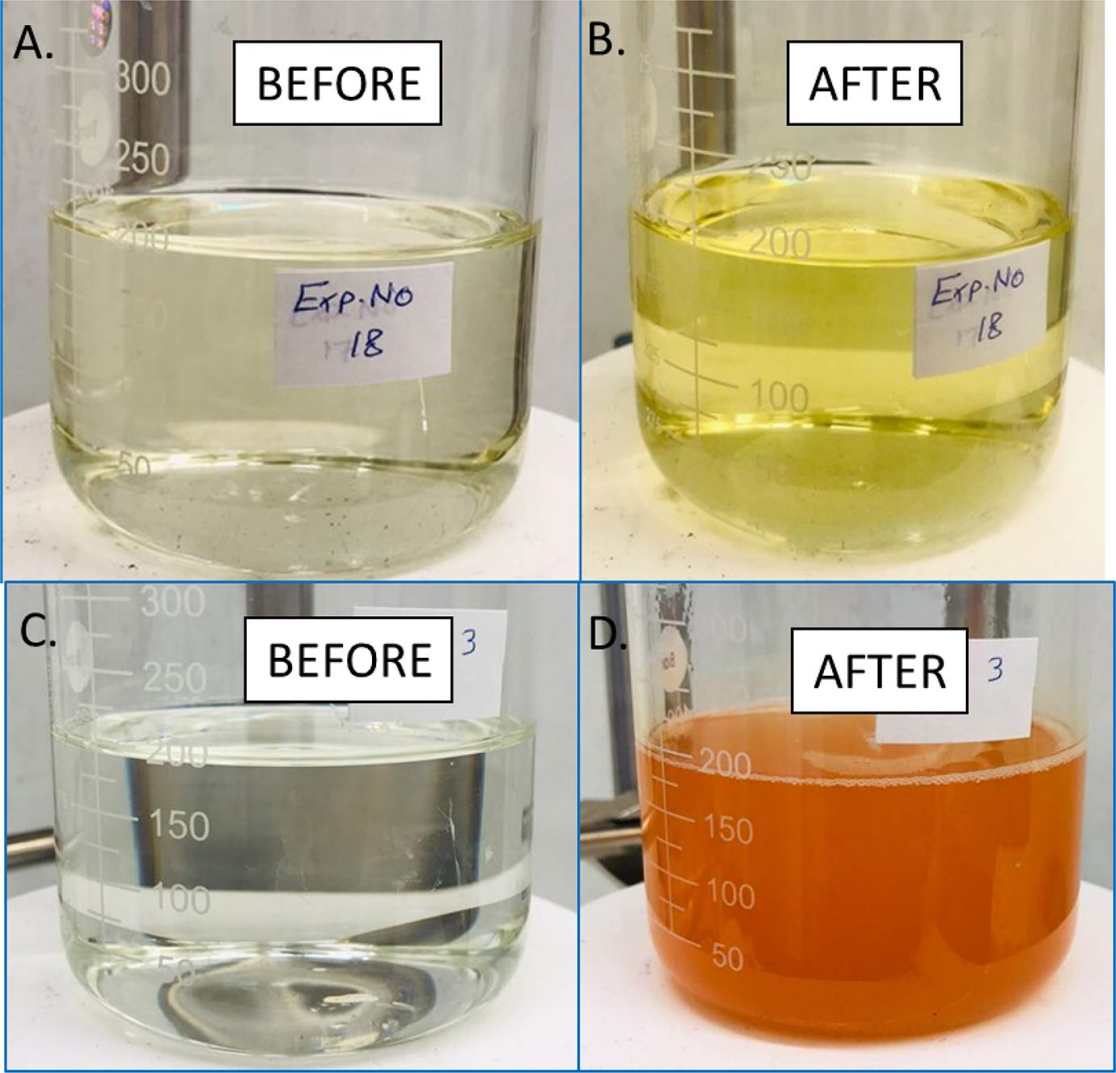
Electrolyte solution of GLDA 5 wt.% + DTPAK_5_ 5 wt.% (top) and DTPAK_5_ 10 wt.% + K_2_CO_3_ 9 wt.% (bottom) . The conditions for the top are (1000 rpm, current density 5 mA/cm^2^) while for the bottom (2000 rpm, current density 50 mA/cm^2^ ).

The dissolution results indicate that the GLDA based formulations, shown in Table 1, which has acidic or neutral pH, has the highest pyrite dissolution over other evaluated formulations (Fig. 6). The pyrite dissolution resulted in a change in the electrolyte color, as in Fig. 7 the reddish color observed after the experiment, which might be indicative of effective Fe^2+^ to Fe^3+^ formation and subsequent mass transfer away from the electrode surface. The pH of the electrolyte solution of GLDA and DTPAK5 before and after the experiment was 7.64 and 7.67, respectively. Similarly, for the pH of the 10% DTPAK_5_ and 9% K_2_CO_3_ electrolyte (Fig. 7) did not show significant change (pH before = 13.43, pH after = 13.59). In both cases, the pH of the electrolyte solution is above 7, which reduces dissolution due to the possible formation of Fe(OH)_3_. In general, the electrochemical dissolution using green formulations demonstrated superior dissolution results compared to chemical dissolution. The minimum electrochemical dissolution obtained for DTPA5Na formulation was more than seven times higher than the chemical dissolution.

**Figure 8:**
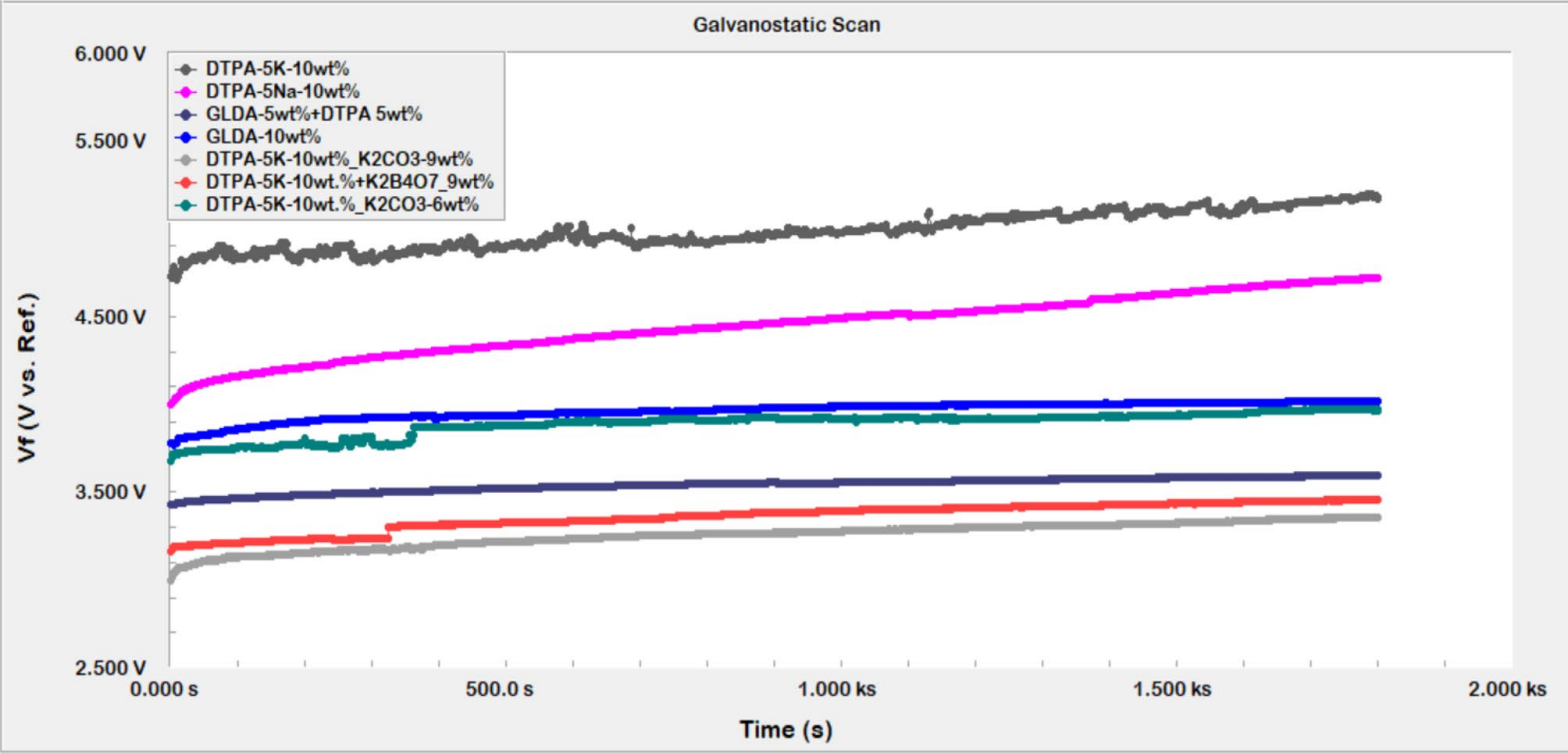
Effect of chemical formulations on the potential–time relationship of pyrite.

From the Galvanostatic test, the measured potential with time was plotted using Echem Analysts Software. The effect of chemical formulations on the potential time relationship was illustrated in Fig. 8. At fixed current density of 5 mA/cm^2^, a noted initial fast increase in the measured potential characteristic of an activation barrier to further electrochemical reactions due to the formation of many oxidation products especially S_8_^26^. Consequently, a slightly increasing potential with time was observed indicative of small barrier for the dissolution of the pyrite due to the small amount of oxidation growth products. The slight increase in potential at this constant current may be considered as indicative of the active electrochemical anodic reactions and their relatively fast subsequent dissolutions with minimum limitations due the small amount of oxidation products resulting in a negligible mass transfer barrier. This would result in minimum effect of the electrode rotation rate as it was noted that the dissolution amount was not affected by the electrode rotation rate when we applied 50 mA/cm^2^ where a higher amount of oxidation products is formed due to the large current density applied, which emphasizes again the electrochemical nature of the dissolution process.

The voltage–time data, V(t), was fitted to the following equation:

6 $$V-{V}_{o}={V}_{ss}\left(1-{e}^{-kt}\right)$$

where V_o_ and V_ss_ are the initial and steady state values of the voltage, respectively and k is the rate constant. The rate of buildup, k, as well as the other model parameters are given in Table 2; the results indicate that the combined formulation of DTPA 10 wt.% with K_2_CO_3_ 9 wt.% had the highest value. This implied that the rate of buildup is very fast with this formulation corroborating what was observed from our previous works^8,27^. It is observed that both the second and third highest buildup rates are also DTPA formulations that is DTPA5K 10 wt.% and DTPA-5Na 10 wt.%, respectively.

**Table 2:** Model parameters, Eq. (4), for the different formulations.

Formulation	V_0_	V_ss_	k
DTPA-5K-10 wt.%	4.73	0.16	0.0180
DTPA-5Na-10 wt.%	4.00	0.36	0.0049
GLDA-5 wt.% + DTPA-5 wt.%	3.43	0.11	0.0032
GLDA-10 wt.%	3.78	0.17	0.0069
DTPA10 wt.%_K_2_CO_3_-9 wt.%	3.00	1.00	0.1709
DTPA-10 wt.% + K_2_B_4_O_7_-9 wt.%	3.16	0.42	0.0010
DTPA-10 wt.% + K_2_CO_3_-6 wt.%	3.68	0.39	0.0014

### Effect of current density on pyrite dissolution:

**Figure 9:**
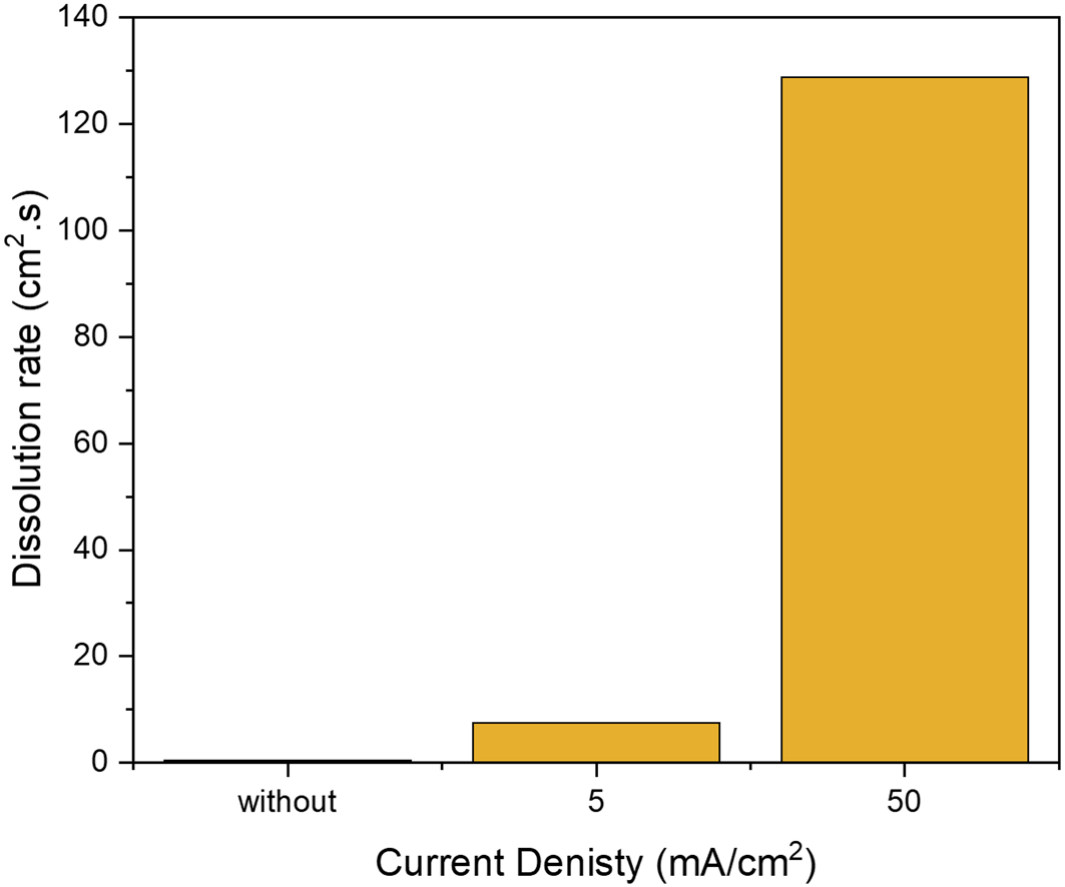
Effect of current density on pyrite dissolution in DTPAK_5_ 10 wt. % + K_2_CO_3_ 9 wt. %.

The impact of the current density on the anodic dissolution of pyrite was studied using three experiments. The first experiment was carried out without applying any current, i.e. at zero current density, while the second and the third experiments were conducted at 5 and 50 mA/cm^2^, respectively. All experiments were conducted at room temperature and atmospheric pressure using DTPA_5_K 10 wt.% + K_2_CO_3_ 9 wt.% solution. The experiments were run for 30 min, followed by the measurement of the concentration of the dissolved iron. The pyrite dissolution increased proportionally with the increase in current density (Fig. 9) as expected.

**Figure 10:**
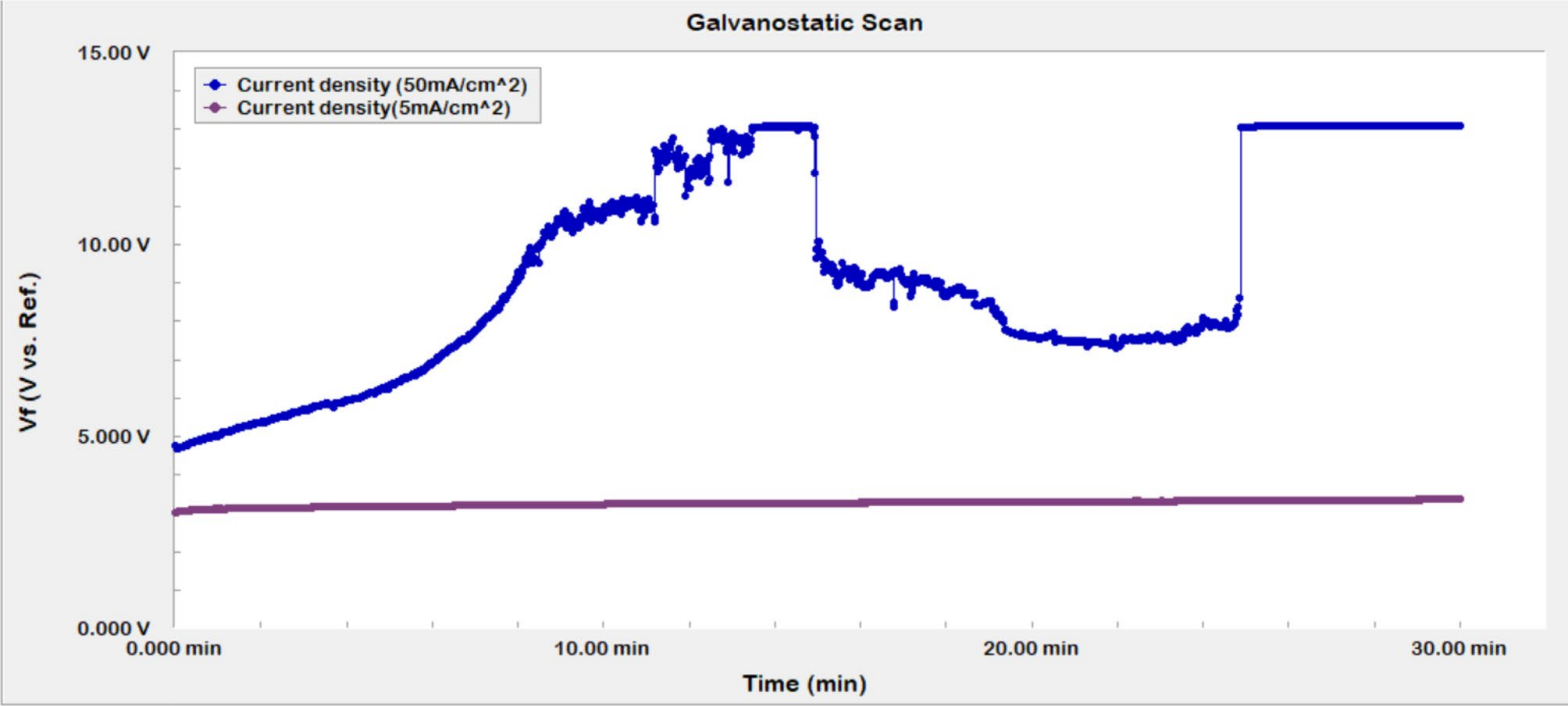
Effect of current density on V(t) for pyrite in DTPAK_5_ 10 wt. % + K_2_CO_3_ 9 wt. %.

From the Galvanostatic test (constant current density), the measured potential with time was plotted using Echem Analysts Software. The effect of current density on the measured potential was illustrated in Fig. 10. At a lower current density of 5 mA/cm^2^, initially, the potential slightly increase due to the formation of anodic oxidation products mentioned earlier, and remain practically unaffected around 3.0 V, where a very small increase with time is noted, which is indicative of the electrochemical nature of pyrite dissolution at high voltages. Whereas, at much higher current density of 50 mA/cm^2^ the formation of large amounts of anodic products was faster due to this very large applied current. This led to a drastic increase in the measured potential, which was close to 13 V where the cut-off potential of the potentiostat is reached followed by a sudden decrease in the voltage, which is indicative of the breakup of the oxidation product.

### Effect of rotational speed on the dissolution rate

**Figure 11:**
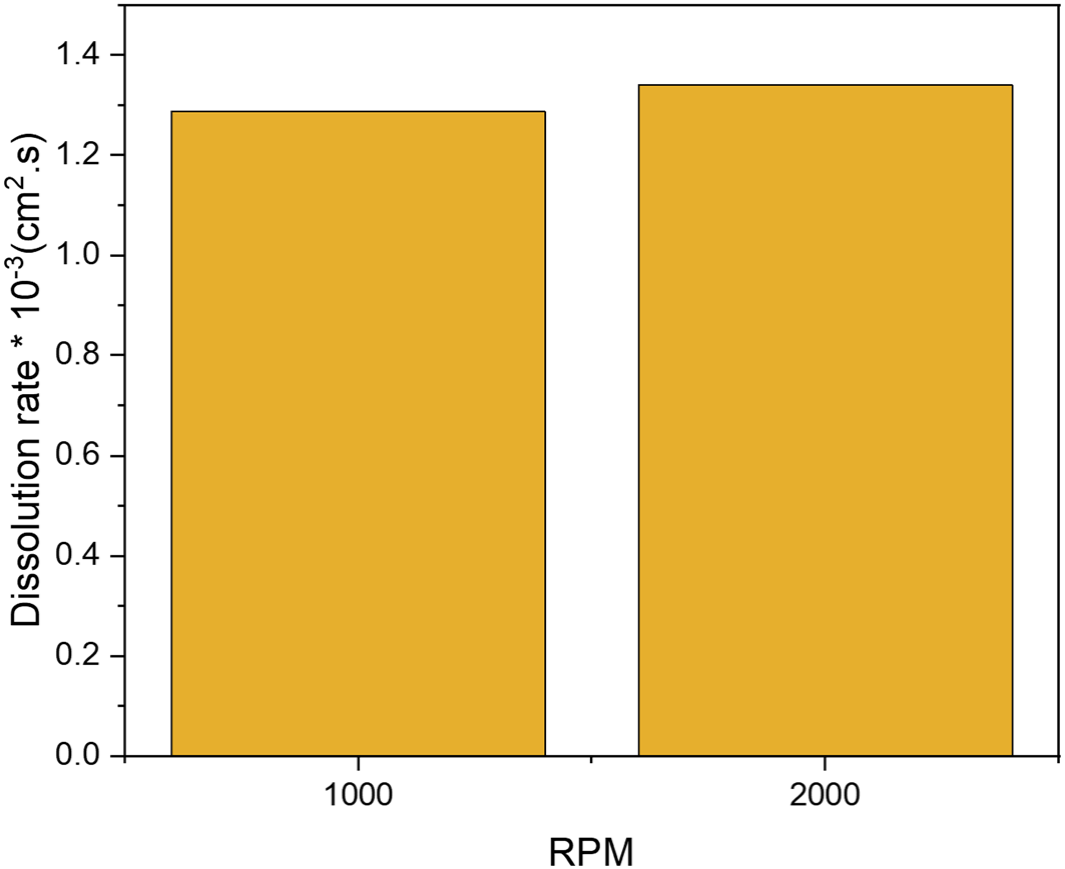
Effect of disc rotational speed on pyrite dissolution in DTPAK_5_ 10 wt. % + K_2_CO_3_ 9 wt. % solution.

The effect of the disc rotational rate on the electro-assisted dissolution was studied at two different rates, 1000 and 2000 rpm. Galvanostatic experiments were conducted using Gamry Potentiostat. The chemical formulation of DTPAK_5_ 10 wt.% + K_2_CO_3_ 9 wt.% solution and a constant current density of 50 mA/cm^2^ were used. A slight increment of 4% of the dissolved iron was noticed when the rpm is increased from 1000 to 2000 as shown in Fig. 11. This increase in rpm has produced almost the same curve during the whole experiment with minimal shift of initial potential of 3 V (vs Ref) compared to a measured voltage of more than 12 V (the limit of the instrument). At lower current density of 5 mA/cm^2^, the potential slightly increases initially and almost reach a plateau. Again, these results could be interpreted in terms of the growth of the layer of the reactant around the surface of the electrode. At low voltage, they do not interfere significantly with the electrochemical dissolution, whereas once a high current is applied, much of the oxidation products are formed and result in a continuously growing voltage until it reached a point where the products are detached (that is dissolution) from the electrode. This process occur suddenly resulting in the sudden decrease of the current.

### XPS analysis

**Figure 12:**
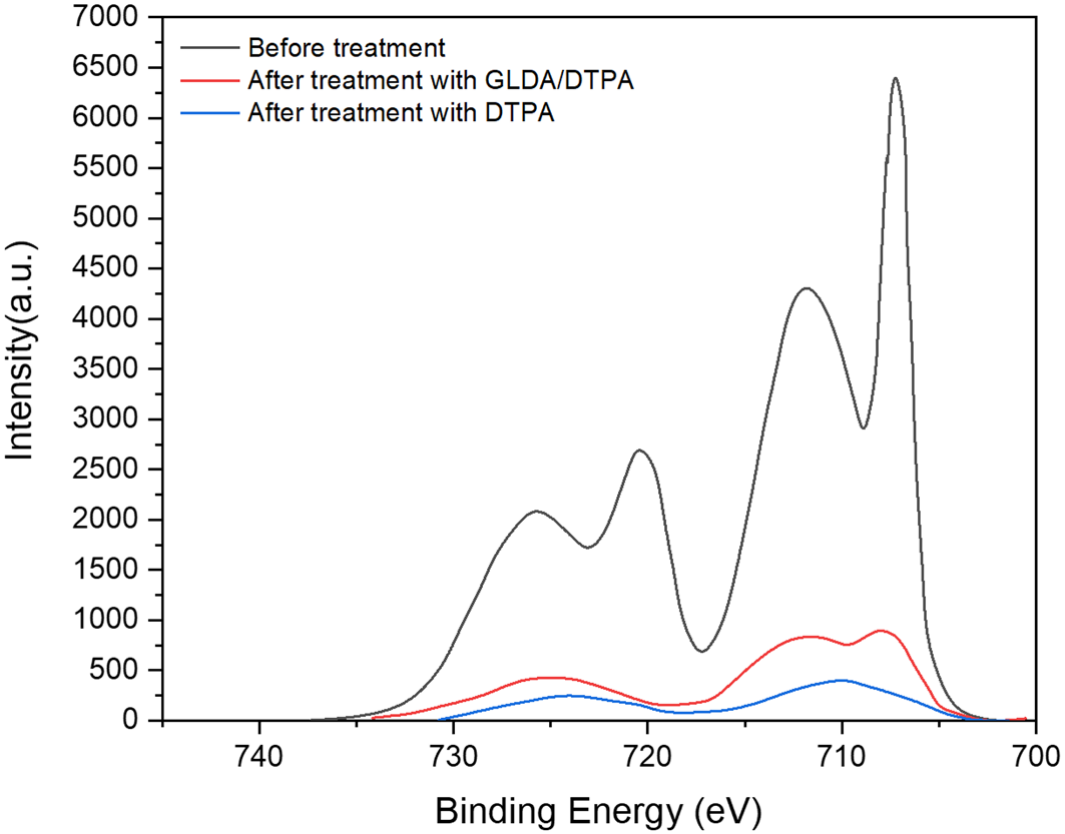
XPS results for pyrite surface before and after treatment with slightly acidic (pH 6.77) DTPA/GLDA formulation and DTPA alone basic formulation (pH 13).

The surface of pyrite electrode was analyzed before and after treatments with basic DTPA formulation (pH  13) and slightly acidic formulation of DTPA/GLDA (pH 6.77) to cover the range of pH used in this study. Figure 12 illustrates the XPS results of pyrite surface before and after treatment with both DTPA and DTPA/GLDA formulations. Before treatment, the peaks at 707.2 and 721 were attributed to Fe^2+^ in pyrite according to previous reports^28,29^. After the electrochemical reaction with both the acidic and basic formulations, these peaks were reduced and new peaks at 711 and 725 are formed due to the electrochemical reaction. These peaks were assigned to the formation of iron oxides^30,31^ on pyrite surface and confirm the oxidation of Fe^2+^ on pyrite surface to Fe^3+^.

There is no doubt that the cost of the electrochemical assisted process of pyrite scale is vital. Nevertheless, a holistic techno-economic analysis is beyond the scope of this work. The cost for the electrochemical process depends on the price of electricity and this varies depending on location as electricity cost varies as it could be null or negative if obtained from renewable resources^32^. Nevertheless, Table 3 shows the cost of chemical formulations in removing 1 kg of pyrite scale. Summarily depending on the energy source of the electrochemical assisted dissolution method. The latter would be more cost effective.

**Table 3:** Cost of removing 1 kg of pyrite using borax and DTPA/K_2_CO_3_ formulations.

Formulation	Reaction rate mg/l-min	The amount formulation needed to dissolve 1 kg of pyrite scale after 30 min in (kg)	Active ingredients (kg)	Water (kg)	cost per $/kg
DTPA + K_2_CO_3_	0.22	196.97	57.12	139.85	564
Borax (K_2_B_4_O_7_)	0.264	138.01	19.32	118.68	103
GLDA	1.824	20.92	4.18	16.74	22

It worth mentioning that, the cost of the removal of pyrite scale calculated here is based only on the chemical cost required however for overall removal cost additional costs should be added. The additional cost varies from one field to another, and includes transportation, field operation, down time and so on.

## Conclusions

Pyrite is one of the most challenging forms of iron sulfide scale that creates a flow assurance problem. Due to the low solubility of pyrite, even when a strong acid such as HCl is employed, alternative solutions have become more necessary. In this work, electrochemical dissolution with effective green formulation inhibiting the formation of H_2_S, is introduced as a possible new approach for removing pyrite scale that forms in the oil and gas tubular system. Unlike inorganic acids, the suggested new formulation does not produce toxic hydrogen sulfide and hence reduce environmental hazards as well further metallic corrosion by H_2_S. The effect of current density, rotational disk rate, and green formulation types on the electrochemical dissolution of pyrite were addressed confirming the electrochemical dissolution aspect of the process at high voltages. The electro-assisted anodic dissolution experiments in the potentiostat and the rotating disk electrode systems have shown specifically the following:

- The total iron dissolved of pyrite using the electrochemical process is more than 400 times higher than the chemical dissolution, at the same conditions, in DTPA/K_2_CO_3_ formulation.
- The dissolution rate increased by 12-folds with the increase of current density from 5 to 50 mA/cm^2^.
- Almost typical trends were observed for potential change with time. A slight increase of the measured potential with time was observed at lower current density of 5 mA/cm^2^ .While at higher current density a rapid increase of potential was noticed due the fast formation of lower conductivity oxidation products that reduces the electrochemical surface and resulted in drastic voltage increase especially at high current densities.
- Acidic and neutral formulations showed better dissolution capability compared to the basic green formulations. However, basic conditions may be preferred in field applications to avoid corrosion in the fluid injection system.
- Besides, doubling the disc rotational rate from 1000 to 2000 did not yield a significant increase in the electro-assisted dissolution of pyrite scale indicating that the electrochemical dissolution is rate controlled with minimum mass transfer limitations.
- XPS analysis confirmed the electrochemical dissolution is mainly due to oxidation of Fe^2+^ on pyrite surface to Fe^3+^.
- Application of electro-assisted dissolution for oil field-scale could make a significant contribution to the scale removal technology. The results herein could serve as a proof-of concept, which could be further developed into an equipment that would be used in oilfield pyrite scale removal.
